# Tailored compliant mechanisms for reconfigurable electromagnetic devices

**DOI:** 10.1038/s41467-023-36143-6

**Published:** 2023-02-13

**Authors:** Galestan Mackertich-Sengerdy, Sawyer D. Campbell, Douglas H. Werner

**Affiliations:** Department of Electrical Engineering, Computational Electromagnetics and Antennas Research Laboratory (CEARL), The Pennsylvania State University, University Park, PA 16802 USA

**Keywords:** Electrical and electronic engineering, Mechanical engineering

## Abstract

Reconfigurable electromagnetic devices, specifically reconfigurable antennas, have shown to be integral to the future of communication systems. However, mechanically robust designs that can survive real-world, harsh environment applications and high-power conditions remain rare to this day. In this paper, the general framework for a field of both discrete and continuously mechanically reconfigurable devices is established by combining compliant mechanisms with electromagnetics. To exemplify this new concept, a reconfigurable compliant mechanism antenna is demonstrated which exhibits continuously tunable performance across a broad band of frequencies. Moreover, three additional examples are also introduced that further showcase the versatility and advanced capabilities of compliant mechanism enabled electromagnetic devices. Unlike previous approaches, this is achieved with minimal part counts, additive manufacturing techniques, and high reliability, which mechanical compliant mechanism devices are known for. The results presented exemplify how compliant mechanisms have the capacity to transform the broader field of reconfigurable electromagnetic devices.

## Abstract (web summary)

Reconfigurability and robustness are two key properties for electromagnetic devices. Here, the use of compliant mechanisms—which are known for their inherent durability, structural stability, and high-accuracy manipulation capabilities in mechanical applications—is introduced for reconfigurable electromagnetic systems.

## Introduction

The comprehensive subject of reconfigurable electromagnetic devices has become the recent focus of active research^1–5^. Reconfigurable antennas form an active subdivision of antenna and communications research primarily targeted at achieving reconfigurability in the RF, microwave, and millimeter-wave frequency regimes. Current reconfigurable antenna solutions can be classified according to their method of achieving actuation. While mechanical, all-electronic, material-based, and optical methods represent the most common approaches to achieve reconfigurability, each can overlap to create new and innovative methods to enable device tunability^1–5^.

The sub-class of reconfigurable antennas are antennas that dynamically achieve an adaptable transformation of their frequency, radiation-pattern, polarization, and/or bandwidth characteristics to enable multiple dynamic functionalities. As such, reconfigurable antennas, compared to their static counterparts, provide engineers with a platform to reduce the number of different antennas required to realize all desired functionalities. One of the first reconfigurable antenna systems was published in 1935^6^. In that work, it was shown that the radiation pattern of a rhombic antenna can be modified by adjusting counterweights which vary the wire element angles, therefore changing the resultant radiation pattern^6^. Considering the needs of emerging technologies and applications such as mobile communications, Multiple-Input-Multiple-Output (MIMO) systems, autonomous vehicles, and integrated space and terrestrial communication networks, doing more with less is key^1,5,7,8^.

The field of reconfigurable antennas can facilitate the reduction of the hardware bottleneck by allowing multi-functions to co-exist within a single antenna. Further advancements, such as, lessened hysteresis between configuration states, long mean time between failures and high-power handling can help maximize performance of reconfigurable antennas. Moreover, it is desired to extend the operational capabilities as these improvements can lead to a lowering of system costs by reducing the number of required onboard components and devices^7,9,10^.

One of the most popular types of mechanically reconfigurable antennas are those referred to as Origami Antennas (OA) as they take their inspiration from the Japanese art of paper folding. Origami antennas have been implemented in extensive ways, ranging from helical antennas capable of switching their radiating pattern polarization^11^, reducing system costs by utilizing paper as a substrate structure^12^, and frequency selective surfaces and deployable reflecting surfaces^13,14^. The biggest drawback of OAs is that specific behaviors and material performance assumptions need to be made in order to realize targeted functionality. Some of these limitations include requiring a sufficiently thin target substrate with negligible elasticity and that the folding section includes motion not along substrate folds. Moreover, if the substrate thickness is increased to improve mechanical rigidity, then the problem of self-avoidance or non-self-intersection starts to play an ever-increasing role^15^. Other limitations of OAs include structures and patterns that are not typically rigid/robust for field deployability and are limited in their stacking and implementation into three-dimensional structures. Also, due to the propensity to use thin, flexible substrates there is minimal force amplification available to create actuation devices^16,17^. Moreover, when origami folds are incorporated into systems such as solar arrays, the complete structure needs to be redesigned to accommodate thicker/stiffer sheets as well as the increase in total thickness that accumulates with each fold^18^ which in-turn negates the flat-packaging advantage and, possibly, the weight reduction targeted by using origami. Lastly, when the incorporation of thin, flexible substrates (with signal or power traces) is used to replace traditional hinges in folded/deployable applications, upon creasing the substrate and traces, they experience deterioration of not only their mechanical integrity, but also their electrical performance, through material fatigue failure and cracking. These types of designs must still utilize additional supports to improve their structural integrity, which in turn increases their complexity and cost^11,12,14,19^. The limitations of thin and flexible substrates with minimal mechanical supporting structures can be seen in the real-world example of the Hubble telescope’s deployable boom and flat panel solar array structure experiencing jitter during orbital day-night crossings^20^. While the Hubble system was not an origami-packed system, it serves as an important lesson for deployable structures utilizing thin substrates. When the implementation of thin substrates with minimal support structure elements or the reliance of folded thin-substrate sections as mechanical supports are all that is employed in the complete design, though stable on earth or in the laboratory setting, when placed into the operational environment, if not fully vetted, they may not meet the final application requirements.

**Fig. 1:**
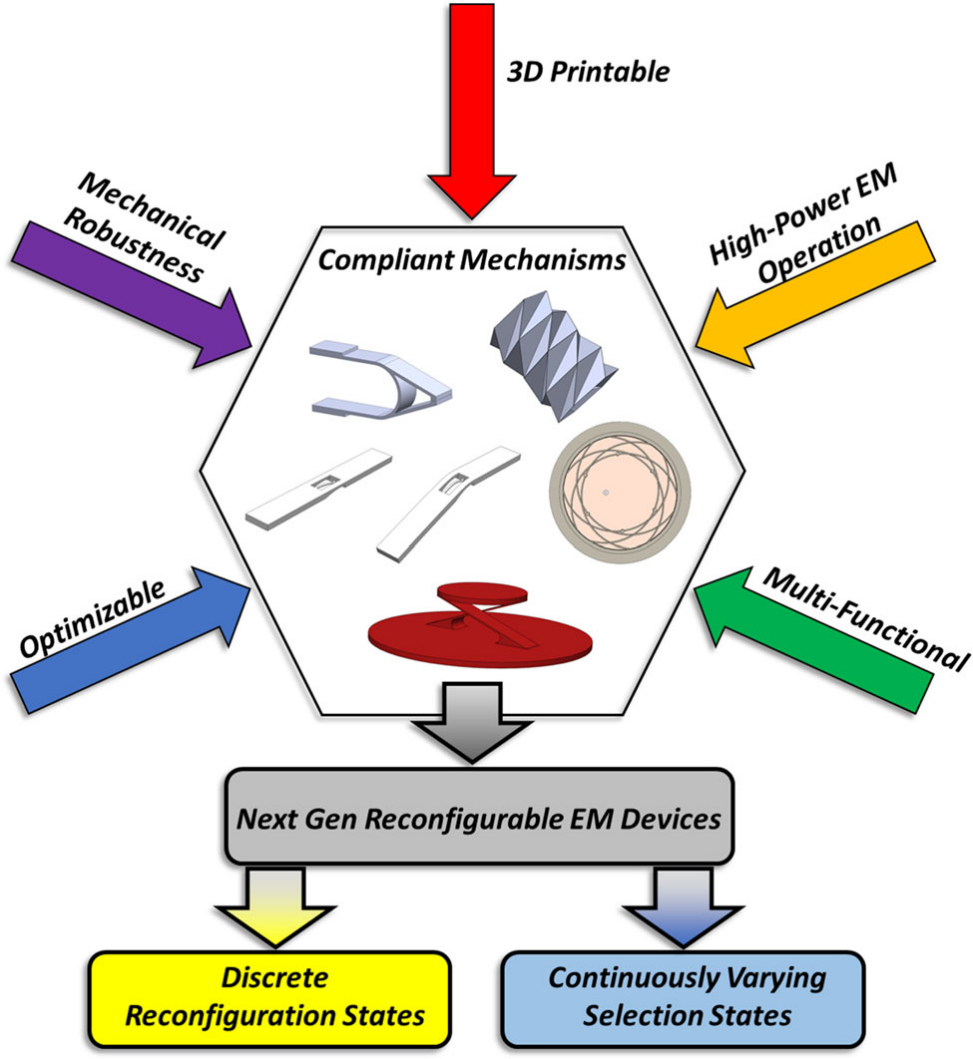
Overview of how the field of compliant mechanisms integrates into reconfigurable antenna and electromagnetic device technology. Compliant mechanisms incorporate the current state-of-the-art mechanical origami methods of reconfiguration as a subset but because of the discrete or continuously varying reconfiguration available to the designer, a completely new framework of reconfigurable electromagnetic designs are achievable. This work aims to demonstrate this new hybrid mechanical-electromagnetic design approach by introducing a compliant mechanism iris-based patch antenna that has the ability to extend the frequency reconfiguration capabilities, all while achieving minimal part counts, robust design for field deployability and simple overall implementation.

To overcome these inherent limitations of not only traditional mechanically reconfigured systems, but also origami-based solutions, as well as other thin-substrate-based methods, an investigation of the current state-of the-art mechanical devices (whether in-plane or out-of-plane motion is required) indicates that there is no work in the broader area of compliant mechanisms integrated into reconfigurable antenna systems. Compliant mechanisms are realized by utilizing a material’s inherent elastic properties to create a desired motion through a controlled deformation. This contrasts with multiple rigid bodies, thin substrates, folding, pins, bearings, and bushings used to develop motion in traditional rigid body kinematic systems, including those used in origami-based systems^21^. Figure 1 describes the role of how compliant mechanisms aim to integrate seamlessly, as well as expand the field with next-generational reconfigurable electromagnetic devices.

Compliant mechanisms can be found in everyday objects such as the single-piece plastic hinges in packaging as well as common all-metal binder clips. Outside of these simplified examples, why are compliant mechanisms desirable? For starters, compliant mechanisms can be made from a single material and as a planar structure but still achieve multi-axis motion. Moreover, they can be designed as a full structure with minimal to no assembly, zero-lubrication required, and their reliability is based on the elastic properties of the material chosen^21,22^. Compliant mechanisms have been proposed for a vast range of disruptive technologies such as low-cost manipulators at nanometer resolution for ultra-precision fiber optic alignment^23^, cervical disc and finger prosthesis^24,25^, surgical endoscopic suturing instrumentation^26^, and overrunning clutch designs^27^. Interestingly, since origami relies on the deflection of flexible materials, it can be categorized as a subset of the broader field of compliant mechanisms^22,28^.

By taking a step back, examining the entire field of compliant mechanisms, and by addressing each of the limitations of origami, we present, in this work, a distinctively different reconfigurable antenna system not previously demonstrated in the literature. The proposed technological innovation regards the integration of robust mechanically reconfigurable structures (i.e., engineered structures through the use of geometry and base material characteristics to allow unique and tailorable mechanical properties and motion), to the passive and active interaction with an electromagnetic source to create transformative variable electromagnetic properties.

**Fig. 2:**
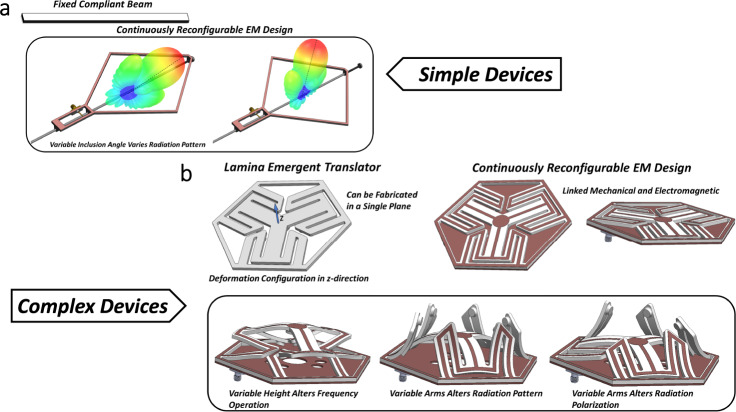
Schematic representations of various compliant mechanisms integrated into reconfigurable antenna concepts: (**a)** reconfigurable antenna with continuously variable pattern options, (**b)** multi-variable reconfigurable compliant mechanism-enabled antenna concept. The simple device shown in (**a**) is reliant on the mechanical deformation of a beam structure. This can be achieved by the continual application of a force to the front section of the device, causing a controlled distortion to the total structure. These types of simple devices can be achieved with compliant mechanisms in either a continuously variable or discrete selection state. The complex device depicted in (**b**) shows how a known compliant mechanism, the lamina emergent translator, could have application to a continually reconfigurable electromagnetic device. Along with the concept of multiple antenna radiation reconfiguration shown, frequency tuning, filtering, or other electromagnetic parameters could be incorporated. Due to the wide variety of choices between discrete or continuous deformation profiles available, novel structures of an antenna or electromagnetic device that can alter dimensions or overall shape based on desired applications are achievable. Shown in Fig. 5 and Supplementary Figs. 1–5, design concepts and simulated results for reconfigurable frequency selective surfaces, phase gradient surfaces or electromagnetic metasurfaces can also be achieved. Such compliant mechanism-based reconfigurable electromagnetic devices could also be integrated into antenna accessories such as ruggedized radome structures that can provide beamforming capabilities to current static antenna setups.

These engineered mechanical structures can then be specifically designed using mechanical optimization techniques for a particular outcome^29–31^. The mechanical optimization results can then be integrated directly into the antenna system where optimization techniques^32,33^ are employed to achieve the required electromagnetic properties. Further work can be directed toward a coupled electrical/mechanical optimization algorithm where topology optimizations of the mechanical and coupled electromagnetic figure of merit target function can be simultaneously maximized. Moreover, utilizing additive manufacturing techniques opens more avenues of novel development of the compliant mechanism structures for electromagnetic applications. Figure 2 showcases just a few of the many possibilities for compliant mechanism-based reconfigurable electromagnetic technologies.

The proposed implementation of compliant mechanisms and other similar structures are robust, can absorb or amplify forces depending on the objective, be designed for specific state selection or continual motion, and can be implemented into stacked structures (with various levels that can perform various functions or reside in states) or as non-symmetric three-dimensional structures, where the vertical implementation does not have to be linked to the horizontal implementation^34^.

In this work, we demonstrate a reconfigurable tailored compliant-mechanism antenna based on a simple circular shorted patch. The compliant mechanism structure is an additively manufactured (3D printed) iris-type model which achieves continuous frequency reconfiguration across the band of 3.5 GHz to 6.2 GHz at a radiation efficiency greater than 80%. In addition, the supplementary section introduces a number of original compliant mechanism-based electromagnetic structures.

## Results

### Reconfigurable antenna selection and compliant mechanism

The patch and monopole antenna represent two of the most popular antennas in use today. However, the operational frequency of such antennas is directly related to their geometry and size which generally is fixed upon fabrication. While there are methods to reduce the total size and achieve reconfigurability in patch and monopole antennas, including loading with voltage-controlled components (e.g., varactors, variable inductors, or pin diodes), these techniques can have limited implementation due to the associated increase in required control circuitry, poor quality factors, limited dynamic range, nonlinearities, and power handling concerns specifically when exposed to high-power microwave conditions^35–38^.

While it is true that the speed of reconfiguration will be slower for mechanical reconfiguration means (hundreds of nanoseconds for all-electronic in comparison to hundreds of microseconds for mechanical), it is known that in specific deployment scenarios, all-electronic reconfiguration is either not easily achieved or completely impractical.

For example, in high-power applications, where the incident electric fields can cause the active circuit elements (e.g., varactor diodes) to short circuit either across the solder joints or internally, a full failure of the switching element can be triggered. Another example where high fields can cause failures would be improperly designed biasing lines for the switching element or scenarios where the reconfigurable component power supply may overdrive the electronic switching element. These power surges or improper biasings can drive the electronic switch to its non-linear region causing eventual failure or corrupted data transmission. Mechanical means can provide lower power consumption by utilizing high efficiency, low-cost stepper motors, and a high electrical isolation since the majority of the controller components can be located below the ground plane^9,10,35,39–43^.

It should also be noted that if the electronic component is included in-line with the transmission line components to achieve reconfiguration, real-word small capacitance effects of the switching elements need to be accounted for due to a parasitic resonance that becomes evident. These effects are typically not seen in simulation as they are assumed to be negligible and therefore ignored. These real-world effects can be accounted for but now a separate cascaded microstrip passband filter is required. Another real-world effect of these switching components is the insertion losses, typically in simulation they are assumed to be zero, but most practical components have losses around 0.5 dB or larger. Mechanical means, specifically compliant mechanism-based means, can be fabricated with extremely low-loss dielectric materials reducing the required biasing lines, cascaded filters, component losses, and other real-world losses typically not accounted for in simulation^9,10,35,39–43^.

To demonstrate the capabilities of the newly described field of compliant mechanism-based reconfigurable antenna topologies, a custom multi-leaf spring compliant revolute iris was designed to guide six shorting pins to travel, in contact, along the bottom ground plane and the upper radiating patch element. The compliant mechanism selected allows continuously variable resonant frequency reconfiguration to be achieved by the antenna through a simple mechanical action. Antennas with a high-gain and conical or monopole-like radiation pattern are typically desirable for wireless ground, vehicular, and mobile communication systems^44^ where high-speed reconfiguration is not necessary but environmental ruggedness is highly desirable.

The antenna presented demonstrates the concept of compliant mechanisms and their vast implementation possibilities. If this specific antenna was to be implemented with the associated control system, it would primarily be located below the ground plane so as to not interfere with the electromagnetic operation. The manually operated lever would be replaced with either a gear/pulley or linkage-based system located away from the critical internal components. These types of gear/pulley/linkage and stepper motor activation methods have demonstrated themselves to be robust as there are implemented currently in other mechanical-based systems.

### Design and optimization procedure

Patch antennas operating in their first or fundamental radiation mode have the main beam directed perpendicular to the antenna (i.e., broadside configuration). To rapidly evaluate their performance, conventional circular microstrip patch antennas can be represented as a dielectric-loaded circular cavity model. This technique is popular as it allows the derivation of the resultant electric and magnetic fields inside the cavity as well as provides analytical expressions for predicting the far-field radiation patterns. The cavity model also yields a useful expression to predict the approximate resonant frequency corresponding to a particular circular patch radius, substrate thickness and dielectric constant. In addition, it is known that microstrip patch antennas operating in higher-order modes can generate patterns with nulls in the broadside direction similar to a classical monopole antenna radiation pattern. To excite these higher-order modes, multiple shorting pins arranged symmetrically at a specific radius around a center-fed circular patch antenna can create the traditional monopole antenna type radiation pattern^45^. Using the dielectric-loaded cavity model and higher-order operational modes, the approximate resonant frequency of the system in relation to the circular patch size can be readily determined.

When comparing to all-electronic means of frequency reconfiguration, the common Field Effect Transistor (FET) switches, pin-diodes and varactors^43,46–49^ either obtain a binary option of frequency selection e.g., for WiFi dual-band operation^46^, or if implementing a simple control circuitry, the bandwidth of frequency operation is increased by less than 10%^47^. Some implementations double the frequency range of operation but are binary solutions and alter the radiation pattern^48^. A variable frequency antenna utilizing varactor components was able to achieve continuous frequency reconfiguration, but the operational frequency bands are limited by the bias-voltage thresholds of the varactors. This can be seen in the designs achieving a range of 6.16–6.47 GHz^43^ or dual-band antennas that operate at 1.68–1.93 GHz and 2.11–2.51 GHz for the low-band and high-band modes, respectively^49^. This work presents a reconfigurable compliant mechanism antenna (CMA) with stable operation from 3.46 GHz to 6.37 GHz. Achieving nearly an octave of continuously variable frequency reconfiguration demonstrates the ability of compliant mechanisms to transform the field of reconfigurable electromagnetic devices.

The target goals were to achieve a high-gain conical or monopole-type radiation pattern for a frequency regime that exceeds current state-of-the-art all-electronic solutions, while offering other performance advantages such as structural robustness and actuation simplicity. For the design presented, the overall radiation pattern can be modified by the inclusion of slots, parasitic elements, or even a second conductive layer with a metasurface to perform beam manipulation.

The design procedure, including the optimization methodology, is described as follows: First, the ground plane dimensions, feed method, and various top patch sizes for the antenna were parametrically adjusted to achieve the desired operational frequency range. Next, the approximate total package height, including the top patch location and supporting structure for the radiating patch, were varied parametrically to determine their total impact on the system as well as to obtain required reconfigurable compliant mechanism design constraints. Then the largest patch geometry was modeled using commercial full-wave simulations tools and the shorting pins were designed based on readily available Commercial Off The Shelf (COTS) parts and existing manufacturing practices. A parametric sweep of the number of shorting pins and their total body radius was performed, giving insight to the effects of shorting pin inductance in series with the static capacitance of the patch antenna model. A determination of the best shorting pin radii and their radial locations along with the antenna feed point were considered at this step to achieve the desired operational frequency range and optimal feed matching conditions. An independent mechanical model of the chosen compliant mechanism, based on approximated material properties, was simulated to assess the shorting pin displacement profile. These displacement paths were then incorporated into the electromagnetics model as well as key radio frequency (RF) material properties to increase its accuracy. Lastly, parametric sweeps of the entire structure were performed to determine which setup is the most robust to manufacturing tolerances while maintaining the desired electromagnetic performance.

### Fabrication procedure

**Fig. 3:**
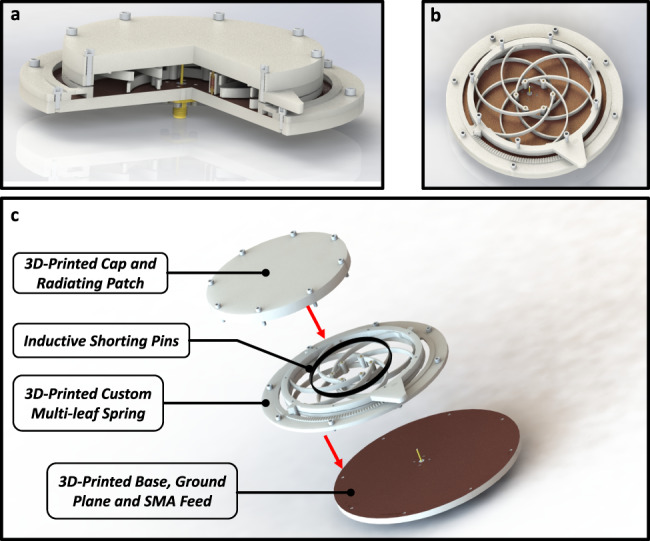
Renderings of the proposed reconfigurable compliant mechanism antenna (rCMA). Shown in **a** is a cutaway view of the entire rCMA revealing the custom shorting pins, the overlapping iris arms to allow motion and the feed pin in contact with the top radiating patch. In **b**, the top plate of the CMA is removed showing the orientation of the shorting pins in the smallest diameter configuration. Image **c** is an exploded view of the rCMA where the three main components are visible, namely the 3D-printed cap and radiating patch, the custom 3D-printed iris compliant mechanism and shorting pins, and the 3D-printed base with the ground plane and SMA antenna feed.

**Fig. 4:**
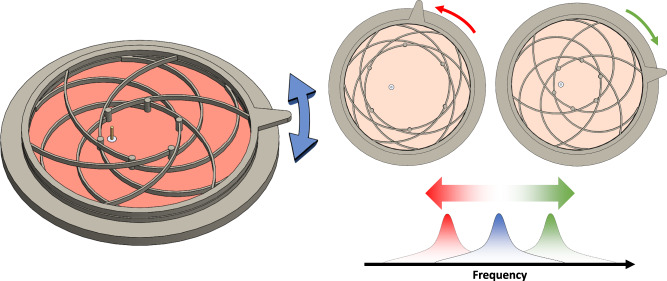
Overview of the proposed reconfigurable CMA operation. Shown is the simplified operation as well as targeted antenna characteristics of the reconfigurable CMA system. As the top section is rotated, the arms displace, thereby changing the location of the shorting pins symmetrically around the center of the patch antenna. This changes the resonant frequency of the system creating a operational frequency band for that configuration. Because the system as a whole has continuous motion it has continuously varying frequency reconfiguration. This motion is repeatable and due to the fixed rotation limit as well as the elastic properties of the material, the displaced arms rebound back to their original state, but also allow a high cycle lifetime.

During the design process, a suitable material needed to be chosen that accommodates not only the required RF properties (low-loss across the large range of frequencies), but also the required mechanical properties to achieve consistent operation without fracturing. The 3D printable material Verowhite from Stratasys was chosen due to its combination of acceptable RF properties and favorable mechanical properties^50^. The three separate components (exploded view shown in Fig. 3c) designed to create the entire antenna were additively manufactured, and copper plates were bonded to the surface to metalize the ground and radiating patch regions. Figure 4 and Supplementary Movie 1 demonstrate how the multi-leaf spring compliant revolute joint motion reconfigures the antenna’s frequency response. The main source of loss to the antenna from the 3D-printed device is the rigid top support structure. In simulation, the loss has been accounted for by including it in the parameter variables. The compliant structure inside the resonant cavity area is minimal due to targeted reconfiguration design, so the impact of the material loss is small.

**Fig. 5:**
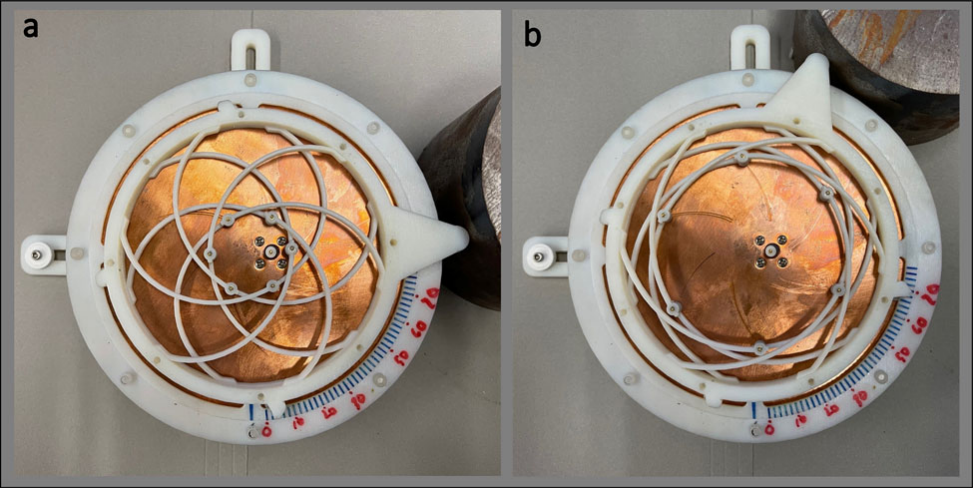
Fabricated reconfigurable CMA with the top radiating patch section removed to observe the motion. Shown in **a** is the relaxed configuration, and **b** is the extended configuration. The repeated path that the shorting pins take along the copper ground plane is clearly visible in the photographs.

Custom pins were fabricated based on the optimal dimensions found from RF simulations. The inductance values that achieve the best matching for all the configuration scenarios are directly correlated to the internal and external radii of the pin shaft, as well as the commercial spring-pin dimensions. The required six pins were fabricated by incorporating standard COTS spring contacts in custom turned copper rods and press-fitted together. After 3D printing, the entire structure was assembled using nylon 4-40 screws. It can be seen in Fig. 5, with the radiating patch removed, that the shorting pins travel along the ground surface when the outer ring is manually actuated.

### Measured results

The final antenna weight, as tested, is 270 g. To put this value in perspective, a simple commercially available 2.4–2.5 GHz antenna with a radome is stated to weigh 40 g and 83 mm in diameter^51^. If we consider a commercially available wideband antenna (1.5 GHz through 4 GHz) we see that the antenna weighs over 0.4 kg^52^.

The weight presented is without the reconfiguration control system, but any reconfigurable antenna will require auxiliary power systems, secondary control boards, and based on the method to achieve reconfigurability, EMI considerations. The advantage of the conical beam or monopolar-like radiation pattern mechanically reconfigured antenna for specific wireless ground, vehicular, and mobile communication systems^44^ is that the reconfiguration methodology is easily ruggedized for harsh environment conditions while the majority of components can be located below the ground plane. On the other hand, electronic varactor and switch-based systems will require complex circuitry and EMI considerations before implementation.

**Fig. 6:**
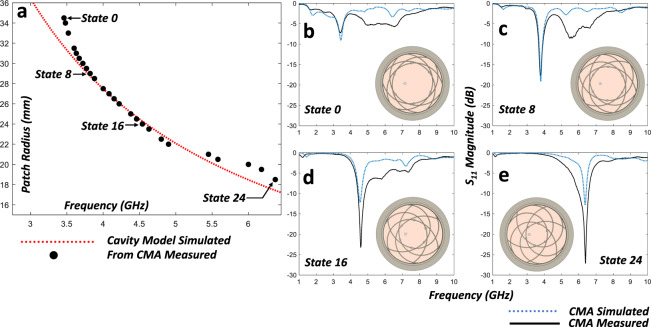
Measured *S*_11_ and resonant peaks compared against the estimated circular patch radius based on the dielectric-loaded cavity model. Shown in **a** is the ideal calculated patch radius corresponding to the given frequency with the traditional dielectric-loaded circular cavity model (red curve) compared against the measured resonant peaks extracted from the *S*_11_ data (black points). The dielectric-loaded circular cavity model is bounded by Perfectly Magnetic Conducting (PMC) walls, as well as two Perfect Electric Conducting (PEC) surfaces on the top and bottom. Shown in **b**–**e** are four shorting pin states, labeled in (**a**), and the comparison of measured S_11_ magnitudes versus simulated results. The cavity model is used to determine the dielectric loading as well as the maximal travel constraints of the shorting pins to achieve frequency reconfiguration. A full-wave Finite Element Method (FEM) model is utilized to tune the shorting pin parameters, total heights and verify the estimated dielectric loading values.

The measured *S*_11_ (i.e., return loss or reflection coefficient) for four chosen configuration states is shown in Fig. 6b–d. Figure 6a shows the comparison of the circular cavity model approximation of the circular patch size to the measured resonant frequency magnitude minimums of the fabricated compliant mechanism antenna. The *S*_11_ results as well as the radiating patch sizes produced by adjusting the shorting pin locations agree very well with the predicted results. It can be seen in some cases presented, that the resonant magnitudes are lower than in the simulated case, which is believed to be attributed to the losses within custom spring-pin manufacturing as well as losses due to a lower-than-expected pressure contact force of the shorting pins along the copper surfaces.

**Fig. 7:**
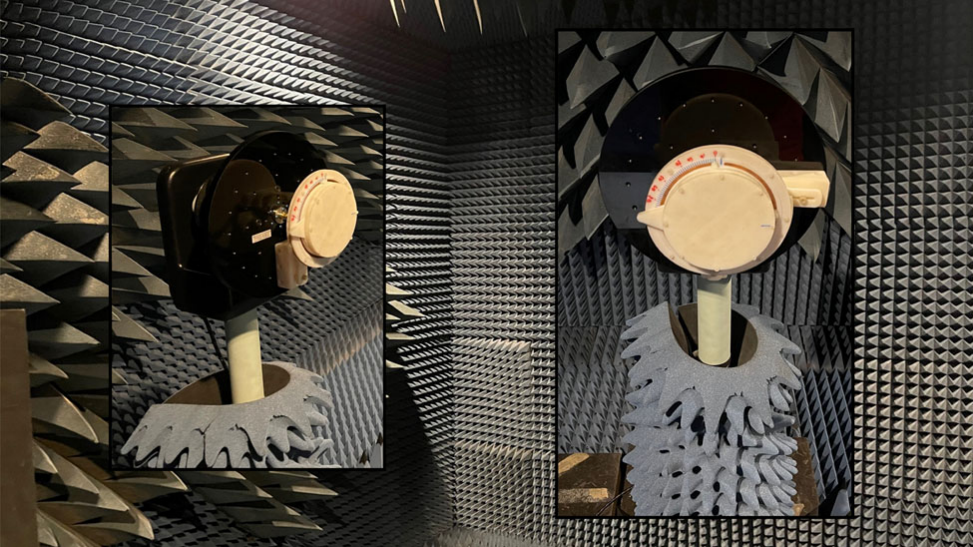
Photographs of the fabricated device. The reconfigurable CMA prototype mounted on the measurement tower roll plate in the anechoic chamber as an isometric view, image on the left, and a front-on view, image on the right.

**Fig. 8:**
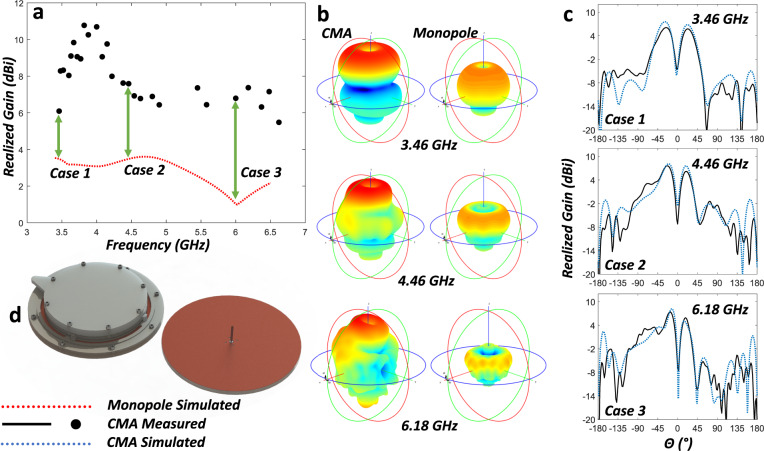
Simulated and measured antenna gain performance, including a static monopole comparison. In **a**, the reconfigurable CMA measured realized gain results clearly demonstrate higher gain than a traditional quarter-wavelength monopole antenna (image of both for comparison depicted in (**d**) showing similar total sizes) across the operational frequency band of both antennas. The simulated monopole antenna performance (shown in **a**, **b**) was optimized for best results at a single height. A quarter-wavelength monopole operation is determined by its height so it does not allow any reconfigurability. It was chosen as the baseline for the comparison due to the similarity in radiation pattern shapes and total package volume. Three independent cases have been isolated and in (**b**) the rCMA and monopole simulated radiation pattern (color and size scaled per realized gain values) are presented at the three case frequencies. It can be seen that the reconfigurable CMA outperforms the static monopole in realized gain, as well as pattern shape retention. In **c**, for the three cases chosen, the (φ = 0) simulated and measured (co-polarization) results for the reconfigurable CMA are compared and demonstrate good agreement with simulation, indicating the simulated 3D radiation patterns shown in (**b**) are accurate and can be expected in application. These measured results serve to demonstrate the enhanced performance capabilities.

**Fig. 9:**
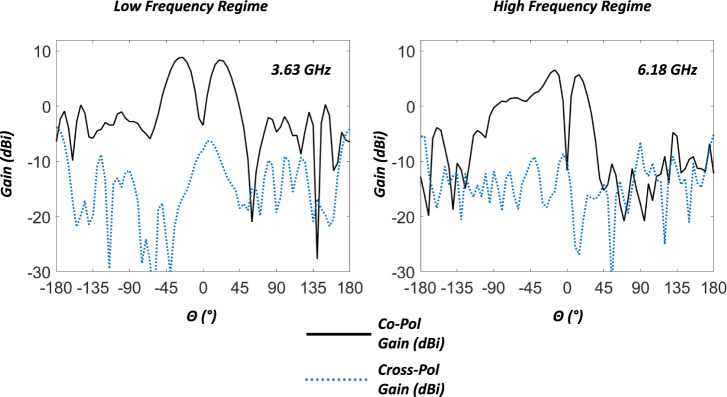
Measured antenna co- and cross-polarization performance. The image on the left represents the antenna’s performance for the co-polarization as well as the cross-polarization at the low-frequency end of the operational state. It can be seen that around the main beam the reconfiguration does not introduce cross-polarization. The image on the right indicates the antenna’s performance at the high-frequency end of the operational state. Once again, the reconfiguration method does not significantly alter the antenna’s performance from the intended polarization.

Figure 7 is an image of the full antenna mounted to the anechoic chamber (ETS-Lindgreen) tower adapter plate. Each independent configuration state was selected by manually rotating and aligning to the desired angle. Friction between the shorting pins, ground plane, radiating plate, and the guide channels of the rCMA held the selected rotation angle. The radiation pattern was then captured and stored for processing. The far-field patterns, shown in Fig. 8, agree well with simulated predictions. Realized gain in the three cases presented are 6.1 dBi at 3.46 GHz, 7.6 dBi at 4.46 GHz, and 7.4 dBi at 6.18 GHz. Maximum realized gain of 10.8 dB is achieved at 3.82 GHz, but as demonstrated in Fig. 9a realized gain doesn’t drop below 6 dBi until 6.62 GHz.

Since the measurements agree well with the simulations, we can confidently conclude that the total antenna efficiency is greater than 80% across the band. The cross-polarization is presented in Fig. 9. It can be seen that the cross-polarization level is well below the co-polarization component for extremes of the antennas operating range. Similar performance is achieved throughout the band. This demonstrates that the proposed reconfiguration method has negligible impact on the radiation properties of the antenna in terms of generating any undesired cross-polarization.

These results demonstrate that the excellent antenna feed match across the various frequencies, achieved by incorporating compliant mechanisms, allows significantly increased performance throughout a wide range of frequencies. The variations seen in the radiation patterns (Fig. 8c), where certain configurations have slightly higher or lower realized gain in comparison to the simulation, is believed to be attributed once again to manufacturing errors and the clamp force altering the expected resultant impedance of the custom shorting pins.

The main operational behavior can be attributed to the radially symmetric shorting pins perturbing the field distribution beneath the radiating patch due to their shunt inductive effect^53–55^, which increases the gain. It is also known that the fields in the region under the top-loaded area can be expressed as a summation of the modal functions weighted by associated expansion coefficients and verified through full-wave Method of Moments (MoM) and wire-model methods^56,57^. It can be considered that the antenna is operating closer to an end-loaded monopole or higher-order microstrip patch than a traditional monopole with similar physical heights.

These types of antennas have been extensively studied and have shown similar performance to simulations with high radiation efficiency and realized gains on the order of 7.5 dB that can be realized. Moreover, for top-loaded monopole antennas a maximum radiation angle of around 25–30 degrees is achieved^53–57^. These properties are all consistent with the presented reconfigurable CMA.

Table 1 presents a comparison of notable reconfigurable origami antennas with the rCMA introduced here. Due to the vast reconfiguration parameter space for antenna characteristics, Table 1 is limited to mainly a comparison to frequency reconfiguration of origami-based antennas. Also included is a single example of polarization reconfiguration. It is believed that due to the extreme versatility of the presented compliant mechanism method, polarization reconfiguration can be easily incorporated into a new antenna design or even the currently presented devices.

**Table 1:** Comparison with key figures of merit for reconfigurable origami antennas

Frequency of operation (centered around)		Continuous or discrete reconfiguration	Antenna efficiencies (at frequency bands)			Pattern reconfiguration	${S}_{11}$ values (dB) (at frequency bands)		Realized gain (dBi) (at frequency bands)			References
Minimum frequency	Maximum frequency		Minimum frequency	Mid frequency	Maximum frequency		Minimum frequency	Maximum frequency	Minimum frequency	Mid frequency	Maximum frequency	
0.77 GHz	1.23 GHz	Discrete	20%	Not capable	90%	None	−11.6	−13.9	−3.41	Not capable	−0.48	^62^
Near 2.15 GHz	Near 2.35 GHz	Discrete	Not specified	Not capable	Not specified	Yes, monopolar to patch	Near −45	Near −10	Near 4	Not capable	Near 4	^63^
0.65 GHz	1.4 GHz	Discrete	Not specified	Not specified	Not specified	None	Near −24	Near −11	6.50	7.31	6.79	^64^
2.26 GHz	4.67 GHz	Continuous is possible, but antenna not manufactured	Not specified	Not specified	Not specified	None	Near −25	Near −28	Not specified	Not specified	Not specified	^65^
Dual band: 0.9, 2.3 GHz	Dual band: 1.5, 2.4 GHz	Discrete	Not specified	Not capable	Not specified	None	Dual band: Near −16 and near −20	Dual band: Near −23 and near −20	Dual band: 1.1 and 2.32	Not capable	Dual band: 3.28 and 1.98	^66^
Single frequency	0.98 GHz	Discrete polarization reconfiguration	Single frequency	Not specified	Not specified	Yes, right hand to left hand polarization	Single frequency	−13	Single frequency	Single frequency	6.82	^11^
0.77 GHz	1.34 GHz	Continuous	Not specified	Not specified	Not specified	None	Near −12	Interference peak, near −13	Not specified	Not specified	Not specified	^67^
0.80 GHz	1.76 GHz	Continuous	Not specified	Not specified	Not specified	None	Near −11	Near −13	Not specified	Not specified	Not specified	^67^
Around 3.5 GHz	Around 6.2 GHz	Continuous	>80%	>90%	>80%	Not incorporated here	Around −9	Around −25	Around 6	Around 7	Around 6.5	This work

The first origami reconfigurable antenna has two discrete operational frequencies but suffers from poor efficiency^62^. The second origami antenna presented has only slight frequency variation but also allows pattern reconfiguration by switching from a patch antenna pattern to a monopole pattern. When considering the fabrication method of the activating section, the fragile nature of the origami hinge is an area of concern, as it is constructed from heat-shrink film and copper foil^63^. Consequently, any attempts to increase mechanical robustness would eliminate the origami hinge action. The third origami antenna has two discrete states, and it allows higher frequency operation when deployed into its second state^64^. The fourth origami antenna is a simple continuously variable frequency dipole antenna but was not fabricated^65^. The fifth origami antenna is dual-band frequency reconfigurable but also exhibits only discrete frequency selection^66^. The sixth origami antenna presented is a design that maintains good performance as it switches between right hand to left hand polarization^11^, but when considering the materials used for fabrication it would be difficult to advance to a robust design for practical implementation into a fieldable system. Lastly, the thick origami antenna presented was able to achieve continuous frequency reconfiguration, as well as utilizing a robust additive manufacturing material^67^. It should be noted that the fabrication technique to achieve the flexibility is a dual print method that requires extensive testing to determine the lifetime of actuation due to separation of the flexible printed joint from the rugged printed substrate. The compliant mechanism-based antenna reconfiguration presented specifically removes that limitation of the design by utilizing the base materials inherent elastic properties. These can be constrained in various ways for a multitude of materials to achieve extended mechanical lifetimes. It should also be noted that the values labeled with “Near” are inferred from the relevant publications’ figures, and due to the continuously variable nature of the reconfigurable compliant mechanism-based antenna, presented values of “Around” have been used to average the range available based on the configuration state.

It can be seen that the majority of the origami-based antennas do not support continuous reconfiguration methods and are almost exclusively discrete systems. Moreover, origami-based systems also have limited frequency performance ranges due to their inherently discrete nature. The presented compliant mechanism-enabled antenna has not only continuously variable frequency selection, but also exceptional total antenna efficiency as well as relatively high realized gain across the band.

### Compliant mechanisms for electromagnetic devices in harsh environments

Compliant mechanisms are prized for their relative simplicity achieved by reducing the part count and utilizing material properties as the source of motion. The implementation of compliant mechanisms has proven to be robust through proposed applications into rocket boosters for space systems, high-precision stages for scanning electron microscopy or semiconductor wafer alignment devices, and surgical replacements for human cervical disc prosthesis. Specifically for space applications, some examples of current challenges that mechanisms deployed in the harsh environment of space include outgassing of lubrication and subsequent binding of joints, thermal gradients causing joint binding, single point failure modes, and increased mass/weight by increased part count. Compliant mechanisms address these challenges by eliminating key joints that require lubrication by a single continuous material that also in-turn reduces part count, as well as redundancy in actuation is feasible and possible miniaturization can be achieved^21–24,58^. This work aims to show that compliant mechanisms have been overlooked for inclusion in electromagnetic devices by demonstrating with a simple and well-understood antenna model that excellent performance at minimal complexity is possible. It should also be noted that the presented device was fabricated with currently available 3D printable materials. With the advances in additive manufacturing and increasing material options occurring rapidly, further expansive applications are expected to become realizable.

Mechanical devices are subject to rigorous testing to determine their expected lifetime. These same lifetime tests are applied to electrical devices such as capacitors, electrical connectors, and batteries that have charge/discharge cycles, insertion limits, and environmental operating conditions. For reliability of specific mechanical systems, compliant mechanism-based devices have shown to be robust alternative solutions^21,22^.

Materials do suffer from fatigue and plastic deformation if their limits are exceeded. Through proper design and simulation, extensive mechanical operational cycles can be achieved with compliant mechanisms^21,22^, due to the fact that they are specifically created for these operational goals. Capacitor life can be reduced with just a 10 °C increase in temperature (and changes corresponding to internal losses)^59^. Mechanical fatigue testing of electrical switches, copper traces, printed circuit board (PCB) laminates as well as solder joints are typically not evaluated for mechanical robustness, but if a solder joint were to succumb to fatigue failure the electrical switch is essentially rendered in-operable. It has even been noted that PCB laminates (a key material that is typically chosen for electrical transmission properties and not mechanical robustness) are most likely a significant factor that influences the overall reliability of electronic assemblies^60^.

When the aspect of high-power is then also considered, low-loss polymers convert the absorbed energy into heat and if the loss is low enough the heat generated can be radiated easily to the environment. Varactor-based devices, as mentioned previously, suffer from high field enhancement produced by the structure geometry, which in-turn limits their performance and restricts the application space^9,35,39^.

As for the specific material used in this design, it has a dielectric breakdown strength of around 30 MV/m^61^. When investigating the maximum electric fields within the cavity and external to the cavity, the maximum electric fields are seen around the coaxial input pin. The total power handling capability is calculated to be over 28 kW. When the SMA connector is taken into account, the input connector is the limiting component of the design (limited to approximately 500 Watts, dependent on frequency and manufacturer).

## Discussion

The results shown here serve to validate the expansion of the field of reconfigurable antenna technology by incorporating compliant mechanisms. The demonstrated reconfigurable antenna achieves continuously tunable frequency operation across a wide frequency range of nearly an octave. The realized gain across the operational band ranged from 6.1 to 10.8 dBi. In addition, the incorporation of the 3D-printed structure allows a substantial part reduction over other methods of pin reconfiguration, while still maintaining a light weight and low height profile. The removal of varactors and pin-diodes, leads to possible implementation into high-power and radiation hardened environments. Further improvements can be to alter the ground plane into a hexagonal shape leading to high packing efficiencies for antenna array implementations. The use of 3D printing allows multiple material selections and the realization of novel geometries. Furthermore, by engineering compliant mechanical systems with specific dual (mechanical and electrical) properties, the electrical components required can be either reduced or eliminated as well as the number of conventional mechanical components required can be reduced/eliminated.

To further demonstrate the capabilities of compliant mechanisms in electromagnetics, a second design of interest is presented which utilizes the same rotational translation approach as the fabricated rCMA albeit in a differing application. This new design is based on a shorted resonator unit-cell which incorporates the compliant mechanism to realize a reconfigurable phase-gradient metasurface for reflect-array applications. Similar to the coaxial fed patch antenna with shorting pins, the unit-cell model relies on the leaf spring element to achieve continuous reconfiguration. The proposed compliant mechanism approach allows a continuous range of selection states to be achieved but also enables a decrease in complexity as only an inexpensive stepper motor and associated simple driver hardware/software are required to realize reconfiguration. The concept and operation of the proposed reconfigurable unit-cell are described in Supplementary Fig. 1 as well as simulated performance within a periodic boundary. Supplementary Movie 2 demonstrates the proposed actuation of a simple pulley system.

A third design of interest is a reconfigurable compliant mechanism frequency selective surface (CM-FSS) derived from a linearly actuated compliant mechanism. This design is based on the well-understood split ring resonator. When the unit-cell is in the flexed/compressed state, it can be seen that the FSS reflects all the incident energy back to the source (band stop configuration). When it is in its relaxed state, however, nearly all of the incident energy is allowed to pass through undisturbed (band pass configuration). The example fabrication presented utilizes an additively manufactured material as the substrate and though would be suitable for further testing is not the material of choice for field-deployment. For field-deployment, a substrate material such as Polytetrafluoroethylene (PTFE), which is not affected by UV exposure and, importantly, not affected by embrittlement over time, would allow a ruggedized CM-FSS structure to survive harsh environments. A visualization of the operation of this reconfigurable FSS and corresponding simulation data can be seen in Supplementary Figs. 2 and 3.

The final example design of interest consists of a circular waveguide fed dielectric rod antenna with steering reconfigurability achieved through an additively manufactured, bio-inspired, multi-axis variable compliant mechanism. In addition to increased gain and beam-steering capabilities, this design concept can be further extended to achieve dynamic polarization control. Currently, dielectric rod antennas require array configurations and complex feeding networks to achieve steering. With the implementation of compliant mechanisms, the design presented enables a deployable rugged system while providing a simple method of achieving steering without the use of multiple antenna elements and feeding networks. The proposed design and simulation results are shown in Supplementary Figs. 4 and 5.

Lastly, these results and provided exemplary systems offer compelling evidence that the field of compliant mechanisms incorporated into reconfigurable communication and electrical systems may be used to augment conventional technology and enable performance that would otherwise be impractical for current state-of-the-art technology, specifically for harsh environments.

## Methods

### Simulation methodology

Antenna full-wave simulations were performed using the commercially available Ansys Electronics Desktop (formerly HFSS). In order to reduce the computational burden, only the material surrounding the shorting pins was included in simulation. The iris arms are considered electromagnetically small in relation to the full antenna system and could therefore be omitted from the simulated model. The displacement profile of a single iris arm was simulated in Solidworks Static Solver. Then a simplified iris model was 3D printed and the location of the shorting pin in relation to the rotational input was verified. These values were then incorporated within the electromagnetics solver to account for the shorting pin radius displacement at various configurations.

### Device fabrication

The compliant mechanism base structure was additively manufactured on the Stratasys Objet 260 Connex3, using RGD835 Verowhite and SUP706B Support material. The model was cleaned in a high-pressure water cabinet and subsequently tapped for the mounting holes.

### Measurement methodology

Far-field patterns for the reconfigurable compliant mechanism antenna (rCMA) were measured in an anechoic chamber from 1 to 10 GHz. A photograph of the rCMA demonstration unit mounted on the range positioner is shown in Fig. 6. The antenna was characterized in receive mode, where a transmitted signal swept from 1 to 10 GHz collimates into a plane wave that impinges upon the reconfigurable CMA. The two-axis positioner on top of the antenna pedestal enabled the collection of far-field energy in great circle cuts about the forward hemisphere of the rCMA. The range of radiation pattern measurement points in the azimuthal plane were taken from −180° to 180° at 1° steps. The far-field gain was measured by comparing the received signal to that of a 07-18-440-NF/BR wideband horn, using the substitution method. A Keysight N5227B PNA Network Analyzer functioned as the receiver, and the data was processed using the Diamond Engineering Desktop Antenna Measurement software and custom Matlab scripts.

### Reporting summary

Further information on research design is available in the Nature Portfolio Reporting Summary linked to this article.

## Supplementary information

Supplementary Information

Description of Additional Supplementary Files

Supplementary Movie 1

Supplementary Movie 2

Reporting Summary

## Data Availability

All experimental raw data and custom scripts that support the findings of this study are available from the corresponding author upon request.
