# Supplementary material for: Tailored compliant mechanisms for reconfigurable electromagnetic devices
Source: Nat Commun. 2023 Feb 13;14:683. doi: 10.1038/s41467-023-36143-6 (PMC9925788; doi:10.1038/s41467-023-36143-6)
Supplement: Supplementary file 1 — Supplementary Information [file 41467_2023_36143_MOESM1_ESM.pdf]

Supplementary Figures

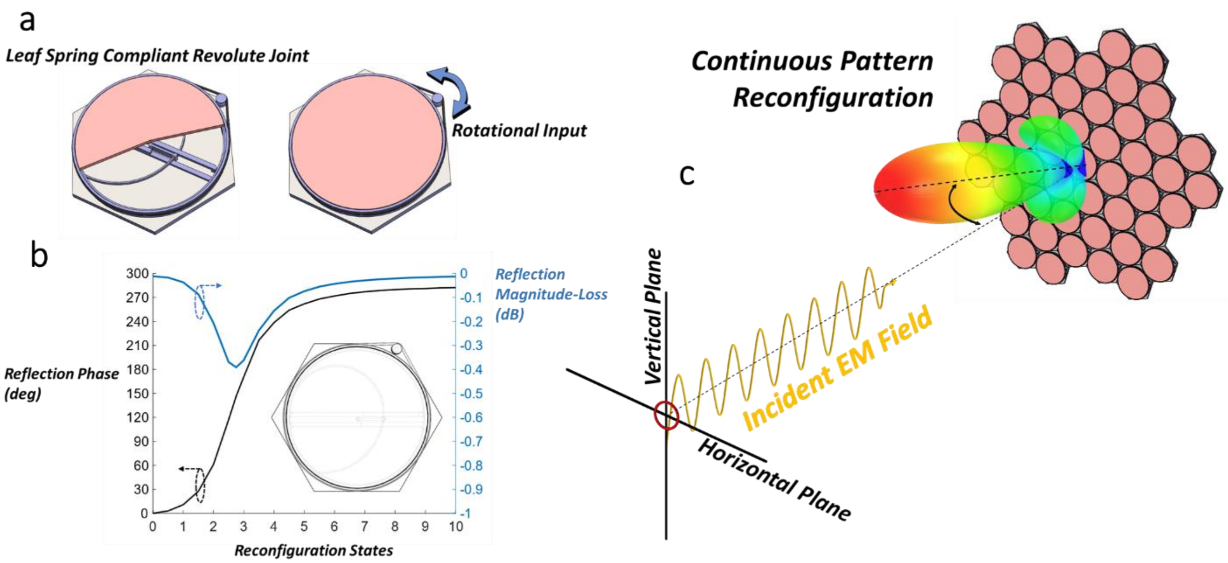

Supplementary Figure 1. Rendering schematic of the proposed compliant mechanism reconfigurable unit-cell. In the work by Gregory, *et al*. (2016) and Gregory, *et al.* (2017), a shorted disk unit-cell demonstrated high-power handling capabilities but also highlighted limitations of implementing the designs they presented into a true field deployable device. The first prototype presented achieved reconfiguration by physically removing the pin manually from the unit-cell. The second prototype used an actuator/relay-based approach where the pins are bridged to ground to achieve the various shorting configurations. The designs were experimentally tested and achieved high-power handling levels beyond any all-electronic design reported in the literature[35-36]. Though successful, a major limitation for field deployment of the first prototype is the requirement of the manual removal of the pin. A limitation of the second prototype unit-cell is the complex circuitry required to actuate the solenoid switching mechanism. With the presented compliant mechanism approach there is not only a continuous range of selection states achievable but also a decrease in complexity as only an inexpensive stepper motor and associated simple driver hardware/software are required to achieve reconfiguration. (a) Cutaway view of the proposed architecture (left) as well as the proposed activation by a simple pulley system (right). The unit-cell is based on a circular conductive disk spaced at a distance away from the ground plane. The shorting pins are allowed to move within a linear track along the surface of the upper conductive disk thereby altering the surface currents across the circular disk. These various surface current configurations achieve a corresponding reflection phase and magnitude. (b) Reflection phase angle and loss as a function of the reconfiguration states for a single frequency. It can be seen that the loss remains less than 0.5 dB for a reflection phase range of nearly 300 degrees. (c) A phase-gradient metasurface reflect-array consisting of a tiling of the unit cells in (a). Here, a normally-incident electromagnetic wave impinging on the reflection surface is being steered by the phase-gradient produced by the independently and continuously varied unit cells. Supplementary Movie 2 demonstrates the mechanical tuning operation of the compliant mechanism.

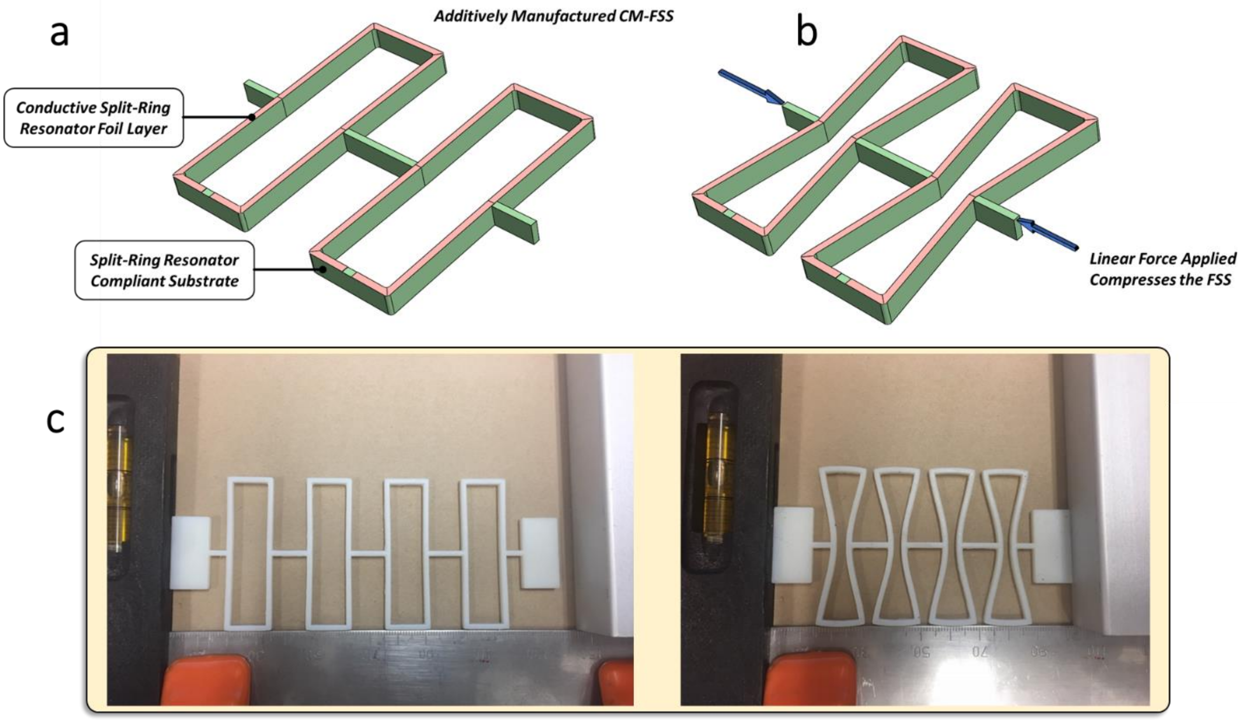

Supplementary Figure 2. Rendering schematic of the proposed compliant mechanism frequency selective surface (CM-FSS). (a) The relaxed or un-compressed state of the additively manufacturable CM-FSS unit cell. (b) The compressed state under the influence of a linear force which is applied along the center and causes a mechanical deformation of the beam element. (c) Views of the fabricated structure in the (left) relaxed and (right) compressed states. The image shown in (c) uses an additively manufactured material as the substrate and would be suitable for further testing. For field-deployment, a substrate material such as Polytetrafluoroethylene (PTFE), which is not affected by UV exposure and importantly not affected by embrittlement over time, would allow a ruggedized CM-FSS structure to survive harsh environments.

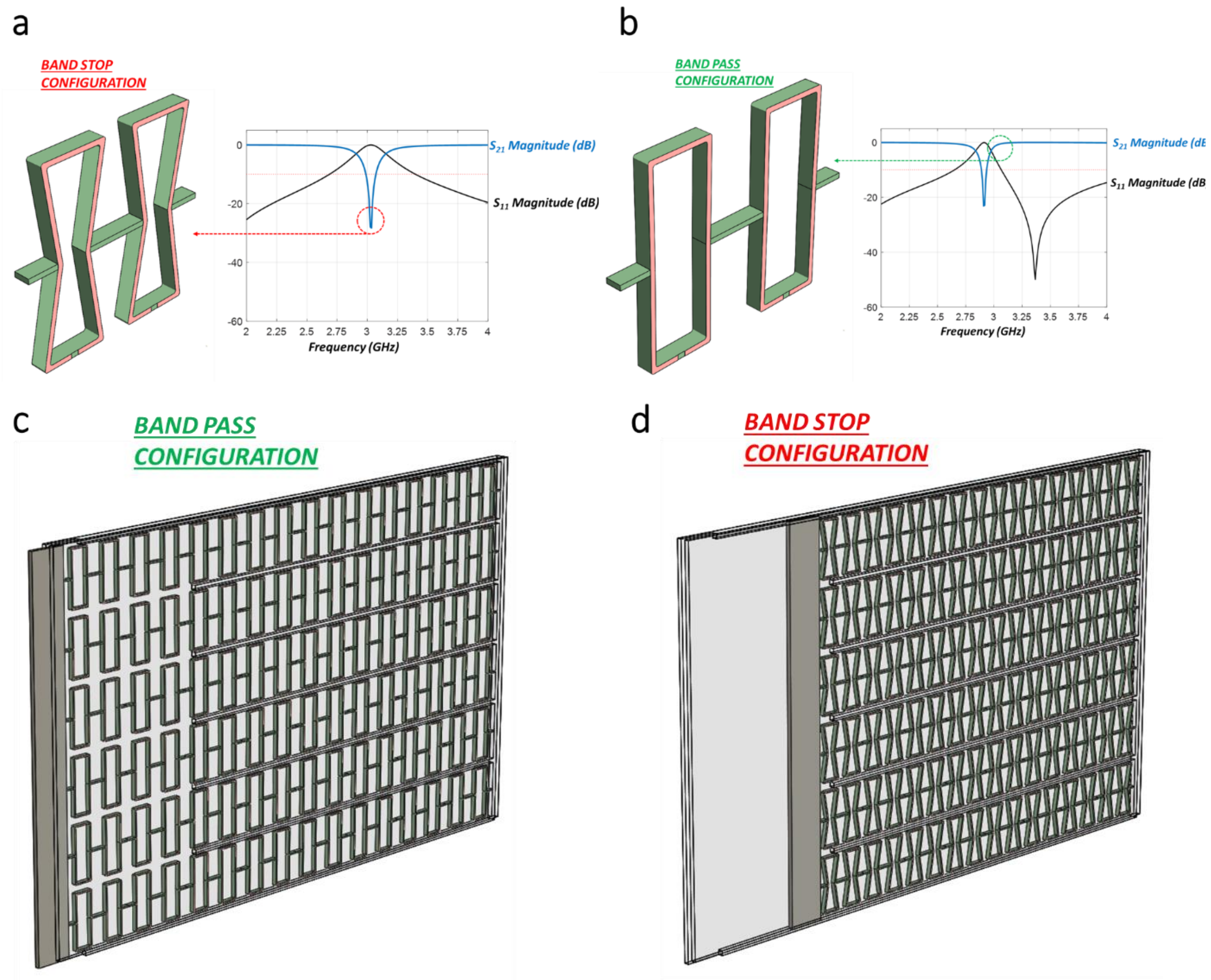

Supplementary Figure 3. Transmission S21 and reflection S11 magnitudes for normally incident waves upon the CM-FSS. (a) The compressed state exhibits single-band filtering performance (*i.e.*, a band stop) at around 3 GHz. (b) The relaxed CM-FSS state demonstrates excellent band-pass performance at 3 GHz. (c), (d) Envisioned full CM-FSS panel with the individual unit-cells captured between two Rexolite guide sheets as well as guide channels. The compression force is applied by a polymer guide plate that can be actuated through linear stepper motors for precise control, or as a discrete system with an on-off configuration.

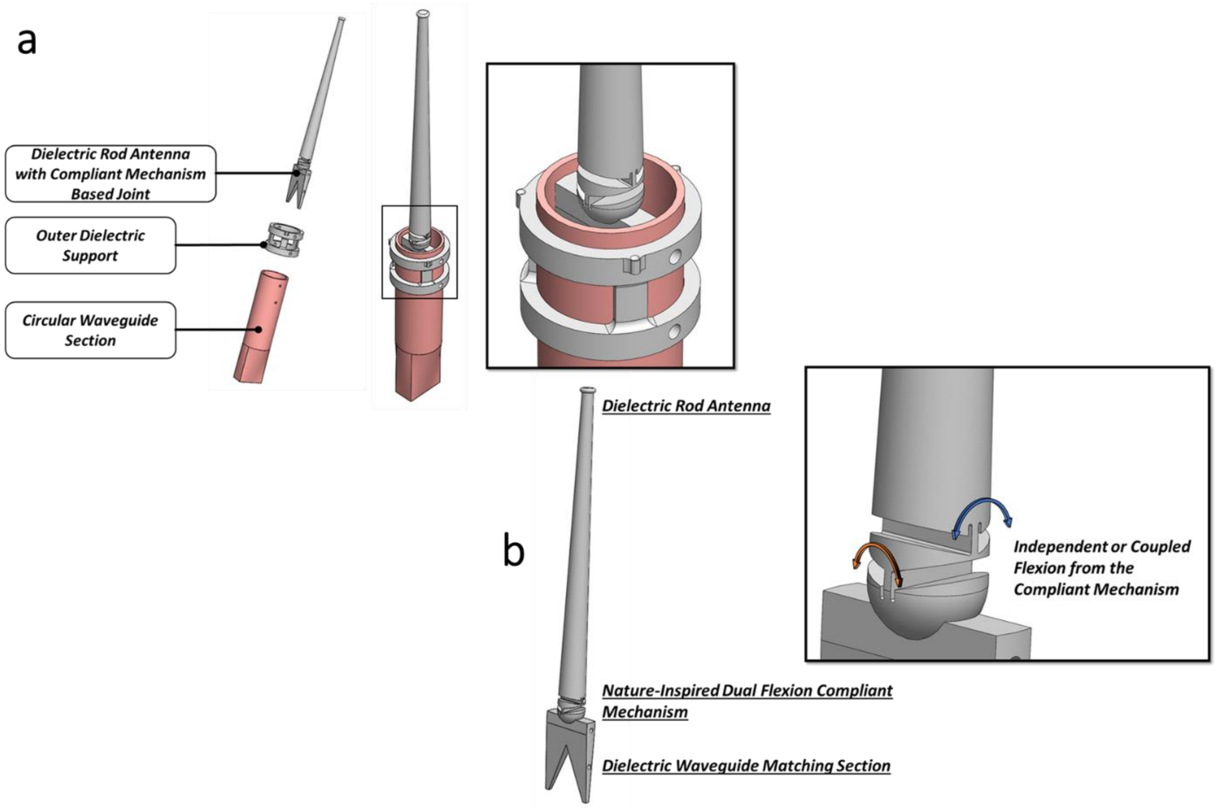

Supplementary Figure 4. Renderings of the proposed reconfigurable compliant mechanism nature-inspired dielectric rod antenna (rCM-NDRA). Currently, dielectric rod antennas require complex feeding array networks to achieve steering. With the implementation of compliant mechanisms, a rCM-NDRA is presented that enables a deployable rugged system while providing a simple method of achieving steering without the use of multiple antenna elements and associated feeding networks. (a) Exploded view of the entire rCM-NDRA revealing the custom waveguide insert that acts as both a mechanical support and serves as the compliant mechanism mounting and the radiating dielectric rod segment while helping to impedance match between the dielectric rod and waveguide. The external section allows the control lines to attach to the dielectric rod. Also shown is the rectangular to circular waveguide conversion section. (b) The dielectric rod antenna system is removed showing in better detail the waveguide impedance matching section as well as the nature-inspired dual flexion compliant mechanism joint. The inset image is a magnified view highlighting how the flexion component operates. If a force is applied along perpendicular to the long-wall section of the joint, it will allow deformation. However, if the same force is parallel to the long-wall it does not allow flexion. This enables both independent movement and coupled deformation if the force is applied at an oblique incident angle relative to the long-wall section of the joint.

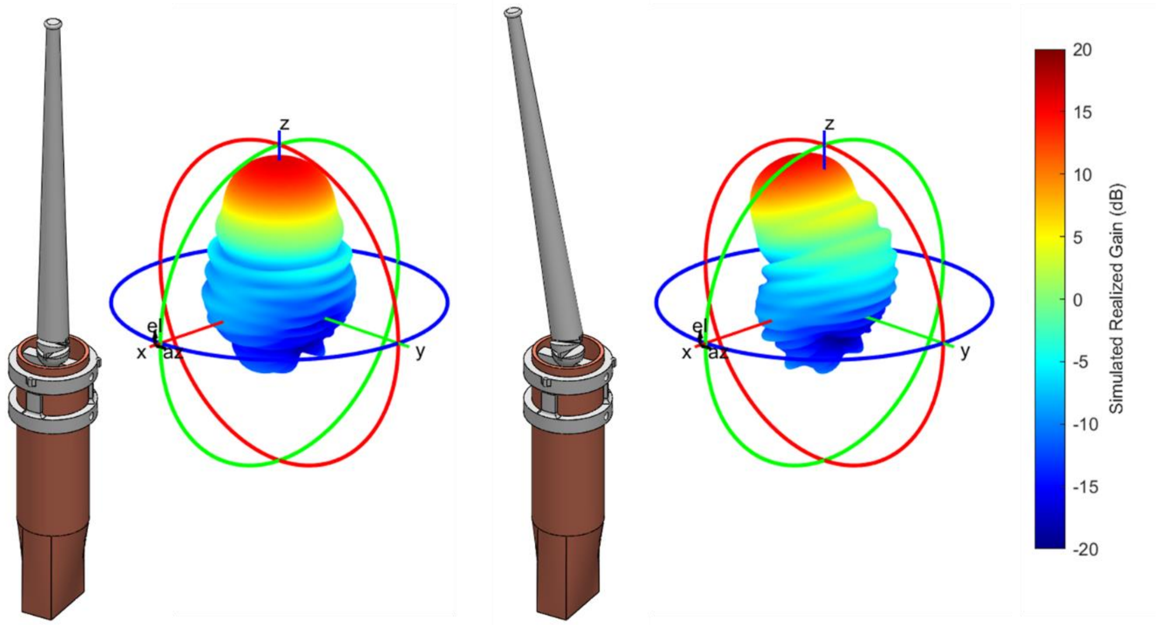

Supplementary Figure 5. Simulated rCM-NDRA realized gain performance in both the broadside and steered configuration. (Left) The broadside configuration showing excellent realized gain performance and beam fidelity. (Right) The steered configuration (single plane steering presented) showing excellent beam pattern fidelity and realized gain performance.
