# Supplementary material for: Tailored compliant mechanisms for reconfigurable electromagnetic devices
Source: Nat Commun. 2023 Feb 13;14:683. doi: 10.1038/s41467-023-36143-6 (PMC9925788; doi:10.1038/s41467-023-36143-6)
Supplement: Supplementary file 2 — Description of Additional Supplementary Files [file 41467_2023_36143_MOESM2_ESM.pdf]

Description of Additional Supplementary Files

Supplementary Movie 1: Demonstration of the lever-based actuation method and subsequent shorting pin motion.

Supplementary Movie 2: Demonstration of the stepper motor and pulley actuation method with subsequent shorting pin motion within the alignment channel.
